# 292. Outcomes of Oral Beta-Lactams versus IV Antibiotics for Gram-Negative Bacteremia in Solid Organ Transplant Patients

**DOI:** 10.1093/ofid/ofaf695.095

**Published:** 2026-01-11

**Authors:** Milen Thomas, Wesley J Hoffmann, William L Musick, Shemual Tsai, Masayuki Nigo

**Affiliations:** Houston Methodist, Missouri City, Texas; Houston Methodist Hospital, Houston, TX; Houston Methodist Hospital, Houston, TX; Houston Methodist Hospital, Houston, TX; Houston Methodist Hospital, Houston, TX

## Abstract

**Background:**

Solid organ transplant recipients (SOT) are at high risk for infections, including gram-negative bacteremia caused by Enterobacterales. While intravenous (IV) antibiotics (abx) are often used, recent studies in immunocompetent patients show that oral abx can be equally effective with fewer adverse events. Data in solid organ transplant recipients is limited, particularly regarding oral beta-lactams (BL).

**Figure:**
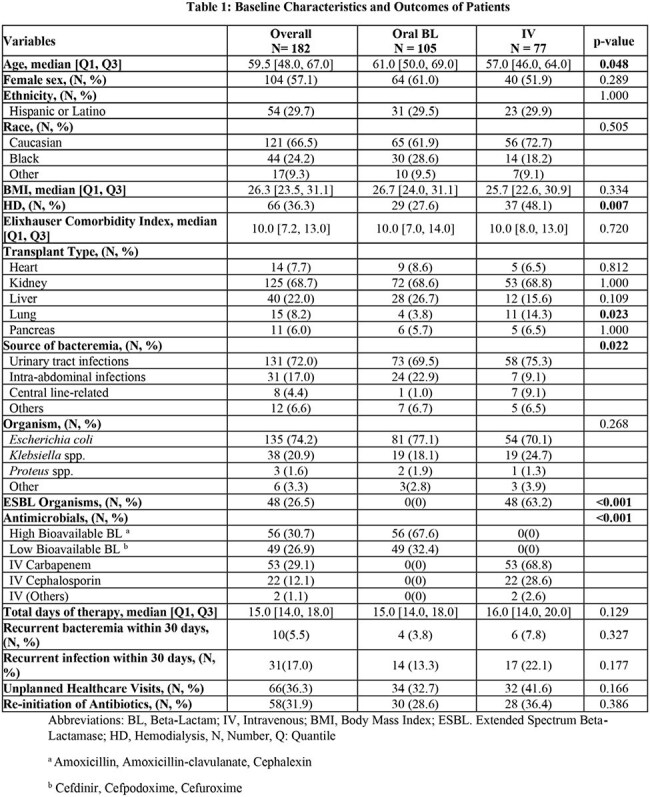

**Figure:**
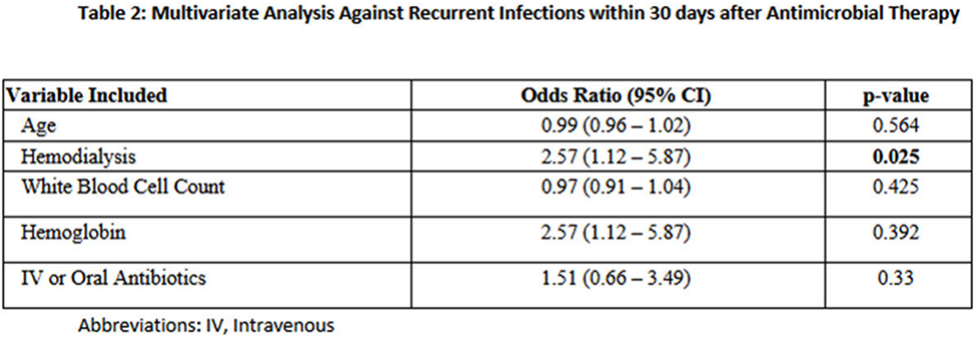

**Methods:**

This retrospective cohort study included adult SOT recipients admitted to the Houston Methodist Hospital System between June 2016 and September 2023 with a first episode of Enterobacterales bacteremia. Patients discharged on either oral BL or IV antibiotics after initial IV therapy were included. Those with deep-seated infections requiring prolonged antibiotic treatment were excluded. The primary outcome was a composite endpoint of all-cause mortality, recurrence of infection, re-initiation of antibiotics, or unplanned healthcare visit within 30 days of antibiotic completion.

**Results:**

A total of 864 bacteremic patients were identified, and 182 were included: 105 in the oral BL arm and 77 in the IV arm. The median patient age was 59.5 years, and 104 of the patients were female. Urinary tract infections were the most common source with 131 cases, and Escherichia coli was the predominant organism with 135 cases. Patients in the BL arm received a median of 6 days of IV therapy before being transitioned. Of these, 37 (35%) in the BL group, 34 (44%) in the IV group met the primary composite endpoint (p=0.22). There were no significant differences in 30-day bacteremia recurrence, 30-day source recurrence, unplanned healthcare visits, or reinitiation of antibiotics between the oral BL group and the IV group. Adverse events and Clostridioides difficile infection rates were low across all groups without significant differences.

**Conclusion:**

Among SOT recipients treated for gram-negative bacteremia, step down therapy with oral BL were not associated with worse outcomes compared to IV abx. These findings suggest oral BL may be a reasonable step-down option in transplant patients with gram-negative bacteremia without deep-seated infections and further prospective studies are warranted to confirm these results.

**Disclosures:**

All Authors: No reported disclosures

